# Beyond the anti-PD-1/PD-L1 era: promising role of the BTLA/HVEM axis as a future target for cancer immunotherapy

**DOI:** 10.1186/s12943-023-01845-4

**Published:** 2023-08-30

**Authors:** Christian Sordo-Bahamonde, Seila Lorenzo-Herrero, Rocío Granda-Díaz, Alejandra Martínez-Pérez, Candelaria Aguilar-García, Juan P. Rodrigo, Juana M. García-Pedrero, Segundo Gonzalez

**Affiliations:** 1 Department of Functional Biology, Immunology, Universidad de Oviedo, Oviedo, Spain; 2 Instituto Universitario de Oncología del Principado de Asturias (IUOPA), Oviedo, Spain; 3 Instituto de Investigación Sanitaria del Principado de Asturias (ISPA), Oviedo, Spain; 4 Department of Otolaryngology-Head and Neck Surgery, Hospital Universitario Central de Asturias (HUCA), Oviedo, Spain; 5 Centro de Investigación Biomédica en Red de Cáncer (CIBERONC), Instituto de Salud Carlos III, Madrid, Spain

**Keywords:** Immunotherapy, BTLA, HVEM, Checkpoint blockade, T cell, NK cell, CD160, LIGHT, Icatolimab, Tifcemalimab

## Abstract

Recent introduction of monoclonal antibodies targeting immune checkpoints to harness antitumor immunity has revolutionized the cancer treatment landscape. The therapeutic success of immune checkpoint blockade (ICB)-based therapies mainly relies on PD-1/PD-L1 and CTLA-4 blockade. However, the limited overall responses and lack of reliable predictive biomarkers of patient´s response are major pitfalls limiting immunotherapy success. Hence, this reflects the compelling need of unveiling novel targets for immunotherapy that allow to expand the spectrum of ICB-based strategies to achieve optimal therapeutic efficacy and benefit for cancer patients. This review thoroughly dissects current molecular and functional knowledge of BTLA/HVEM axis and the future perspectives to become a target for cancer immunotherapy. BTLA/HVEM dysregulation is commonly found and linked to poor prognosis in solid and hematological malignancies. Moreover, circulating BTLA has been revealed as a blood-based predictive biomarker of immunotherapy response in various cancers. On this basis, BTLA/HVEM axis emerges as a novel promising target for cancer immunotherapy. This prompted rapid development and clinical testing of the anti-BTLA blocking antibody Tifcemalimab/icatolimab as the first BTLA-targeted therapy in various ongoing phase I clinical trials with encouraging results on preliminary efficacy and safety profile as monotherapy and combined with other anti-PD-1/PD-L1 therapies. Nevertheless, it is anticipated that the intricate signaling network constituted by BTLA/HVEM/CD160/LIGHT involved in immune response regulation, tumor development and tumor microenvironment could limit therapeutic success. Therefore, in-depth functional characterization in different cancer settings is highly recommended for adequate design and implementation of BTLA-targeted therapies to guarantee the best clinical outcomes to benefit cancer patients.

## Introduction

The immune response is tightly regulated by a complex network of co-stimulatory and co-inhibitory signals mediated by immune checkpoints, which represents an essential mechanism of immunological homeostasis. In the context of cancer, dysregulation of immune checkpoints promotes immune evasion and prevents tumor elimination by cytotoxic lymphocytes, thereby favoring tumor development and progression [1].

The introduction of immune checkpoint blockade (ICB)-based therapies has revolutionized the landscape of cancer treatment within the last few years. The emergence of immunotherapy was supported by the outstanding response rates achieved in several tumors. Moreover, ICB has become a cornerstone for the treatment of tumors classically linked to poor prognosis, such as melanoma, renal carcinoma, or lung cancer, even evolving/turning into first-line therapies in certain cases [1, 2]. Nonetheless, despite these initial encouraging results, response rates to PD-1 and CTLA-4 blocking antibodies do not exceed approximately 20–30%. Furthermore, various tumors, including pancreatic adenocarcinoma or glioblastoma, exhibit complete resistance to ICB-based therapies, thus supporting the urge of finding new immune checkpoints for cancer immunotherapy [1, 3]. To expand the portfolio of targets with emerging checkpoints may also have the potential to benefit a wider range of patients. Within the last years, a plethora of new co-inhibitory molecules has been explored and several of them are currently under pre-clinical and clinical development, including LAG-3, NKG2A or ILT2 [4–8].

This review focuses on comprehensively examining current knowledge on the immunobiology of the inhibitory immune checkpoint BTLA (B- and T-lymphocyte attenuator) and its binding partner HVEM (Herpesvirus entry mediator), as well as currently available evidence supporting the clinical application of the BTLA/HVEM axis as a future target for cancer immunotherapy.

### BTLA is an immunoinhibitory receptor

The inhibitory immune checkpoint BTLA/CD272 belongs to the CD28 immunoglobulin superfamily. This receptor recognizes and binds to HVEM/CD270, a member of the TNF (tumor necrosis factor) receptor family, portraying the first described interaction or crosstalk between both superfamilies. BTLA is broadly expressed in the immune system, mainly on T and B lymphocytes, macrophages and dendritic cells and, at low levels, on NK cells [9, 10]. Likewise, HVEM is expressed on T and B lymphocytes, as well as NK cells, dendritic and myeloid cells, thus suggesting a complex heterotypic interaction between the different immune cell subsets that express both molecules [10]. Regarding T lymphocytes, BTLA expression arises during the positive selection that takes place in the thymus, being higher in CD4+ than in CD8+ T lymphocytes and, unlike PD-1 or CTLA-4, with no expression reported on regulatory T cells (Tregs) [11]. BTLA expression can be detected at low levels in the bone marrow during the pro-B and pre-B phase, whereas it is constitutively expressed on naïve B lymphocytes [12].

**Fig. 1:**
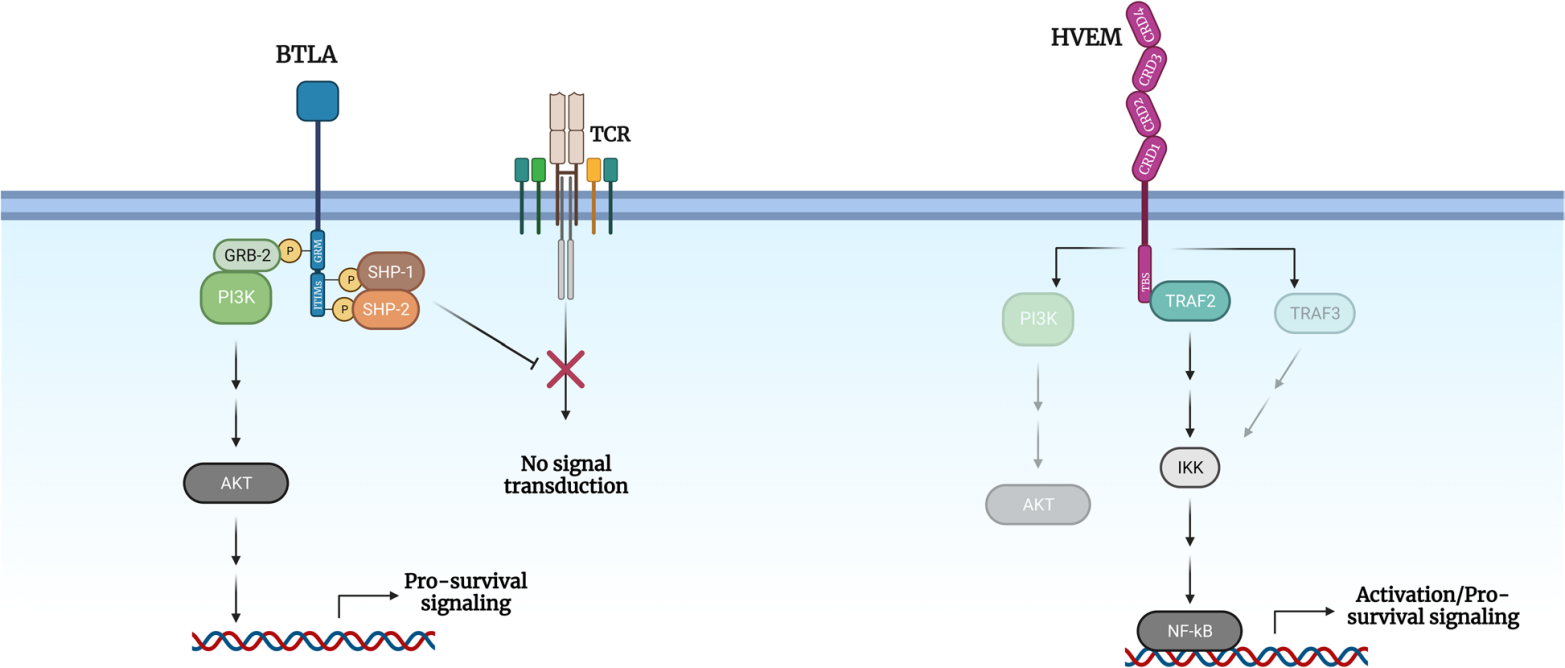
HVEM binding network regulates immune homeostasis. Activation of BTLA signaling leads to the recruitment of SHP-1/2 and negatively regulates T cell responses, whereas GRB-2 promotes survival through the AKT pathway. HVEM-mediated NF-κB signaling leads to enhanced activation status, inflammation, and survival

BTLA signaling negatively regulates immune responses through recruitment of phosphatases 1 and 2 with Src homologous domain (SHP-1 and SHP-2) mediated via two immunoreceptor tyrosine-based inhibitory (ITIM) motifs [13–15]. Noteworthy, BTLA harbors a third domain in its cytoplasmic tail that contains a GRB-2 recognition motif. Recruitment of GRB-2 and the p85 subunit of phosphatidylinositol 3-kinase (PI3K) leads to protein kinase B (PKB or AKT) activation, thus providing pro-survival signaling and bringing forward a dual role of this molecule in the regulation of the immune response (Fig. 1) [15]. Importantly, HVEM functions as a bidirectional molecular switch between activating (CD160, LIGHT, and lymphotoxin LT-α) and inhibitory (BTLA) pathways, depending on the interacting receptor used. Upon binding, HVEM provides pro-survival and proliferative signals through activation of nuclear transcription factor κB (NF-κB) and AKT transcriptional pathways, whereas BTLA attenuates T cell-mediated responses [10, 16–18].

The first evidence for the inhibitory function of BTLA was demonstrated in mice deficient for this receptor, which showed an augmented risk of developing autoimmune diseases, such as encephalomyelitis and whose T lymphocytes displayed increased proliferative capacity as well [12, 19, 20]. In line with these in vivo observations, in vitro studies revealed that BTLA deletion significantly enhanced T cell proliferation upon stimulation with anti-CD3 monoclonal antibodies (mAbs) or peptide-loaded antigen-presenting cells (APCs), while this effect was not observed in the BTLA wild-type (WT) counterparts [12, 20]. Interestingly, BTLA^−/−^ CD8+ T cells showed greater proliferation, despite wild-type CD4+ T lymphocytes exhibited higher BTLA expression [21]. Moreover, larger numbers of memory CD8+ T lymphocytes were detected in BTLA or HVEM KO mice compared to the corresponding WT mice, thus suggesting that blockage of the inhibitory effect of BTLA could increase immune responses to antigenic stimuli [21]. Consistently, treatment of T cells with an agonist anti-BTLA antibody or HVEM-Ig fusion protein led to reduced proliferation and production of cytokines such as IL-2 or IL-4 [22, 23].

Despite previous observations that classified BTLA as an inhibitory immune checkpoint, studies using BTLA^−/−^ graft-versus-host disease (GvHD) mouse models demonstrated that it also plays an important role in favoring T lymphocyte survival. Donor BTLA^−/−^ T lymphocytes exhibited potent alloreactivity compared to WT during the first week of GvHD response, while being unable to maintain the inflammatory response over time [24]. The significant reduction in BTLA^−/−^ T cell count observed after one week could be related to negative signaling mediated by BTLA-HVEM binding as well as the loss of pro-survival signals via the Grb-2 binding domain of BTLA [15, 24]. In agreement, anti-CD3/CD28 stimulated T cells from BTLA KO mice exhibited enhanced proliferation rates compared to WT mice upon treatment with BTLA-Fc fusion protein by HVEM signaling activation [25]. However, it should be noted that no changes in the number of divisions per cell were found, rather a higher number of T cells were able to proliferate. Therefore, BTLA-Fc-mediated HVEM activation induces pro-survival signaling, thus proposing a protective role for BTLA against cell death.

Currently, little is known about the part that BTLA plays on NK cells. Still, upregulation of BTLA on this immune subset has been observed to compete with CD160, an activating receptor with HVEM-binding ability, dampening NK cell-mediated cytotoxicity and possibly impairing immunosurveillance [26].

### HVEM binds to multiple ligands beyond BTLA

HVEM was initially described as the attachment and entry site for herpes virus through binding to the type I and type II gD proteins of the virus [27]. Unlike BTLA, which has an expression pattern mainly restricted to immune cells (both lymphoid and myeloid), HVEM exhibits a broader expression detected in hematopoietic, epithelial and endothelial cells and neurons [13]. This receptor interacts with members of the TNF superfamily lymphotoxin-α (LT-α) and LIGHT, as well as with CD160 and BTLA, which belong to the immunoglobulin superfamily [28]. In addition, SALM5 has been recently described as a novel binding partner for HVEM and their interaction modulates neuroinflammation by inhibiting the myeloid-related inflammatory response in murine models of multiple sclerosis [29].

HVEM binding to its different ligands is mediated by two extracellular topographical regions constituted by four cysteine-rich domains (CRD), known as CRD1/CRD2 (binding site for BTLA and CD160) and CRD2/CRD3 (binding site for LIGHT and LTα) [14, 30–32]. Due to this structural organization, HVEM acts as a bidirectional switch, acting as ligand for distinct co-stimulatory and co-inhibitory molecules, but also being able to activate its own signal transduction depending on the interacting receptor used. This intricate network is crucial for the homeostatic maintenance of immune responses [31].

Signaling through HVEM is mediated by a TNFR-associated factor 2 (TRAF2) binding site located in its cytoplasmic tail. This initial TRAF2 recruitment ultimately translates into NF-κB activation, which controls the transcription of genes that promote activation, inflammation and survival [14]. Despite the disparate location of LIGHT, LT-α, CD160, and BTLA binding sites in the extracellular domain of HVEM, all share the ability to activate the canonical NF-κB pathway [12, 33, 34]. In addition, HVEM stimulation may lead to activation of non-canonical NF-κB pathway through recruitment of TRAF3, although this mechanism currently remains unclear [13]. Similarly, HVEM-mediated signaling has been linked to PI3K and AKT signaling pathway in T cells, although the exact mechanism needs to be fully elucidated [35–37].

#### The TNF superfamily

The HVEM ligands, LIGHT and LT-α, belonging to the TNF superfamily, can be located on the cell surface, or found as soluble forms. While LIGHT has a transmembrane and a soluble form produced by alternative mRNA splicing or proteolytic cleavage of its extracellular domain, LT-α is exclusively assembled as a soluble protein. LT-α homotrimers engage HVEM with low affinity, compared to TNFR1 and 2 receptors. Notoriously, LT-α can also be found on the outer cell surface, throughout its interaction with LTβ, which leads to the formation of a heterotrimer. This complex exhibits lower affinity for HVEM, favoring the interaction of the heterotrimer with LTβ receptor [38, 39].

LIGHT is a cell-surface homotrimer expressed at low levels on hematopoietic cells, including B and T lymphocytes, as well as dendritic and NK cells, although altered expression has been detected upon activation [40]. Indeed, naïve T cell activation leads to enhanced surface LIGHT expression, whereas, in turn, HVEM levels are decreased [41]. By contrast, although high LIGHT levels and low HVEM are expressed on the surface of dendritic cells at basal state,, dendritic cell activation prompts the loss of surface LIGHT and promotes HVEM expression [42]. Altogether, these observations suggest an interaction between dendritic cells and T lymphocytes through the LIGHT/HVEM axis [40–42]. In agreement, several works have reported that LIGHT expression on T lymphocytes and dendritic cells co-stimulates T cell function and boosts cytokine production and proliferation of this immune subset [42–45]. In addition, both soluble and membrane-bound activated T cell-derived LIGHT induce dendritic cell maturation and cytokine production upon HVEM binding [46]. BTLA has been proposed to instruct Treg differentation in dendritic cells. BTLA+DEC205+CD8+ dendritic cells were revealed as the exclusive mediators of CD5 upregulation in T cells, thereby inducing a "tolerizing" effect. By contrast, CD11c+ dendritic cells lacking BTLA failed to increase CD5 expression, leaving immune responses unchanged. BTLA and HVEM functions enable dendritic cells to connect T cell responsiveness with tolerogenic antigens, influencing CD5-dependent Treg cell induction for peripheral T cell tolerance. The absence of these functions impedes tolerance induction, thus emphasizing the joint role of BTLA and CD5 in adjusting T cell responses via tolerogenic dendritic cells [47].

#### The immunoglobulin superfamily

As mentioned above, CD160 binds and activates HVEM through the CRD1 domain [25]. However, this receptor also interacts with MHC class I molecules, both classical (HLA-A, -B and -C) and non-classical (HLA-E, -F and -G) with low affinity [48, 49]. CD160 was initially identified as a glycosylphosphatidylinositol (GPI)-anchored protein displayed on the NK cell membrane. Nonetheless, a transmembrane form derived from alternative mRNA splicing and a soluble form (by the action of metalloproteases) have also been described [50]. Contrarily to HVEM or BTLA, CD160 presents a more restricted expression pattern; the highest levels are detected on CD56dim CD16+ NK cells, although its expression can also be found in γδ T lymphocytes, NKT cells and activated CD4+ and CD8+ T lymphocytes [51–54].

CD160 exhibits a dual immunomodulatory role. On the one hand, the transmembrane form of CD160 exerts an activating role in NK cells, where treatment with an anti-CD160 agonistic antibody led to an increase of the degranulation marker CD107a, as well as recruitment of the CD107a kinase, Src-family p56 (lck) and activation of the Erk1/2 pathway [50]. In agreement, enhanced NK cell-mediated cytotoxicity via CD160 activation was observed upon transduction of the K562 cell line (characterized by the lack of HLA-I expression) with *TNFRSF14* gene encoding HVEM [26]. In addition, CD160 binding to HLA-C induces activation of CD8+ T lymphocytes and NK cells, as well as an increase in the cytotoxic capability and cytokine production of the latter [48, 49, 55, 56]. Whether CD160 actually plays its part as a co-stimulatory molecule remains controversial, since certain studies have suggested an inhibitory activity for this immune checkpoint. Activation of CD4+ T lymphocytes translates into augmented levels of surface CD160, thus competing with LIGHT for HVEM engagement on the surface of APCs [51]. Additionally, in vitro IL-15-mediated NK cell activation favors cleavage of surface CD160 in a metalloproteases-dependent manner, dampening cytotoxic activity of NK cells and CD8+ T lymphocytes by its binding to HLA-I molecules [57].

The enigmatic activity of CD160 in immune function may indicate that this receptor could act as a molecular switch in some cell types. In this scenario, the extracellular domain of the protein involved in the interaction should define whether negative or positive signals are transduced [40].

### *Cis* and *trans* interactions modulate the activity of the BTLA/HVEM axis

**Fig. 2:**
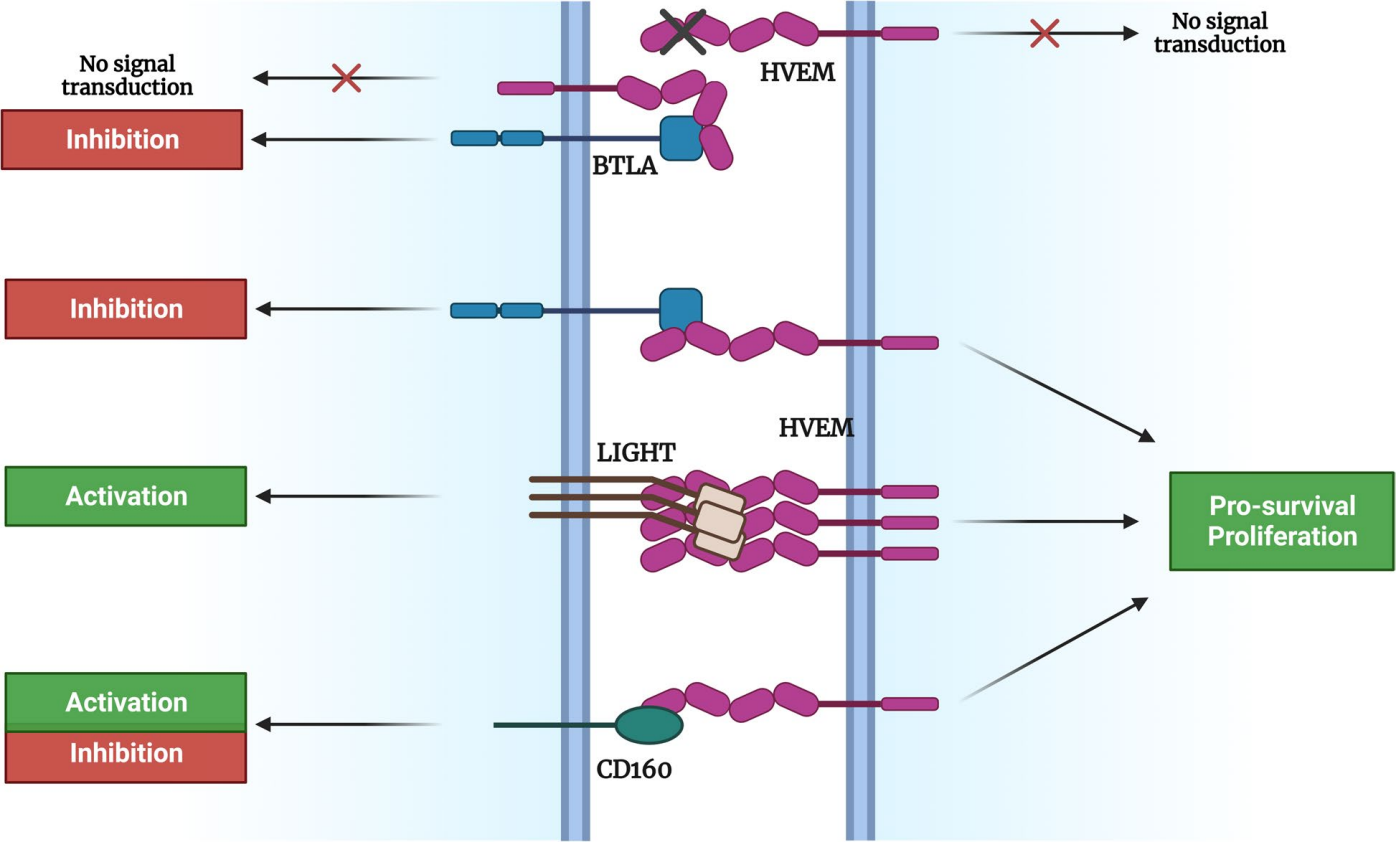
Signal transduction through HVEM and its binding partners relies on the nature of its interactions (*cis or trans*)

Significant efforts have been put into deciphering the complex network established by HVEM. Signaling mediated by HVEM and its ligands depends on three parameters that define the immunobiology of this axis: the ligand-receptor pair involved, whether they are in a soluble or membrane-bound form, and the *cis* or *trans* nature of the interaction (Fig. 2).

As already discussed, immune cells co-express HVEM and BTLA on their surface. In a resting immune system, a *cis* interaction -within the same cell- occurs, leading to the formation of HVEM-BTLA heterodimers. This binding suppresses HVEM-mediated *trans* activating signaling (via NF-κB), therefore maintaining this receptor in a resting state [9, 25, 30, 58–60]. As a consequence of immune activation, the surface levels of another HVEM partner, LIGHT, are transiently increased, a regulatory mechanism that prevents an exacerbated response in a steady state, since LIGHT displays a higher avidity for HVEM as compared to BTLA. Further, upon B and T cell activation, there is also a temporary decrease in HVEM levels and a transitory increase in BTLA expression, causing a dissociation of the *cis* complex and allowing BTLA-HVEM *trans* interactions. This strategy ultimately facilitates LIGHT and HVEM binding, and the consequent activation of the immune response [10, 30]. Although membrane-bound LIGHT can also interact with the HVEM-BTLA heterodimer, it does not induce (or induces at low levels) HVEM signaling cascade. Paradoxically, LIGHT in its soluble form facilitates *cis* interaction stabilization, whereas BTLA or HVEM-Fc fusion proteins cannot even bind to the heterodimeric complex [14]. Despite LIGHT participation in this system, BTLA plays a predominant part in T and B lymphocytes, since it is estimated that the *cis* HVEM-BTLA complex represents nearly 80% of the membrane-expressed proteins [30]. Constitutive HVEM-mediated signaling is detected when co-expressed with LIGHT or CD160, whereas little to no NF-κB pathway activation is achieved upon BTLA-HVEM co-expression, supporting a prevalent BTLA-mediated inhibitory signaling within the *cis* complex. Moreover, BTLA activity has been observed even in *cis* BTLA-HVEM heterodimers. Despite *cis*-heterodimeric complexes prevent *trans* HVEM interactions, recent reports have demonstrated that this binding does not limit BTLA inhibitory pathway [9].

In line with this, BTLA antagonistic antibodies disrupt the *cis* complex, thereby promoting a synergistic effect on HVEM activation via LIGHT [61, 62]. This result suggests that disruption of this complex is mandatory for HVEM activation mediated by the membrane form of LIGHT during the immune response [28]. Nevertheless, Claire Battin et al*.* have recently outlined the potential benefits from targeting HVEM instead of BTLA. Thus, the use of an agonistic anti-HVEM mAb overcomes BTLA *cis-*mediated inhibition and promotes T cell proliferation, activation and cytokine production, suggesting that it may act as both a stimulating and an immune checkpoint-blocking antibody [9]. Still, the therapeutic advantages of targeting BTLA or HVEM deserve further consideration in the context of cancer or autoimmune diseases, where this axis is often dysregulated.

## The BTLA/HVEM axis in solid tumors

T cell exhaustion is a critical phenomenon within the context of immuno-oncology, where the immune system's frontline defenders, including T lymphocytes and NK cells, become progressively dysfunctional and lose their ability to effectively target and eliminate cancer cells. This state of exhaustion arises as a result of chronic antigen exposure and persistent immune stimulation, among others, often leading to altered expression of immune checkpoints in the tumor microenvironment. On this basis, dysregulation of BTLA/HVEM axis has been proposed to play a role in T cell dysfunction using HIV-specific T cells as a model for exhaustion. Antibodies targeting BTLA were found to enhance CD8+ T cell proliferation and cytokine production in response to HIV-1 antigens. In addition, co-targeting PD-1 and BTLA was found to be particularly effective in enhancing responses of exhausted human T cells [63].

**Fig. 3:**
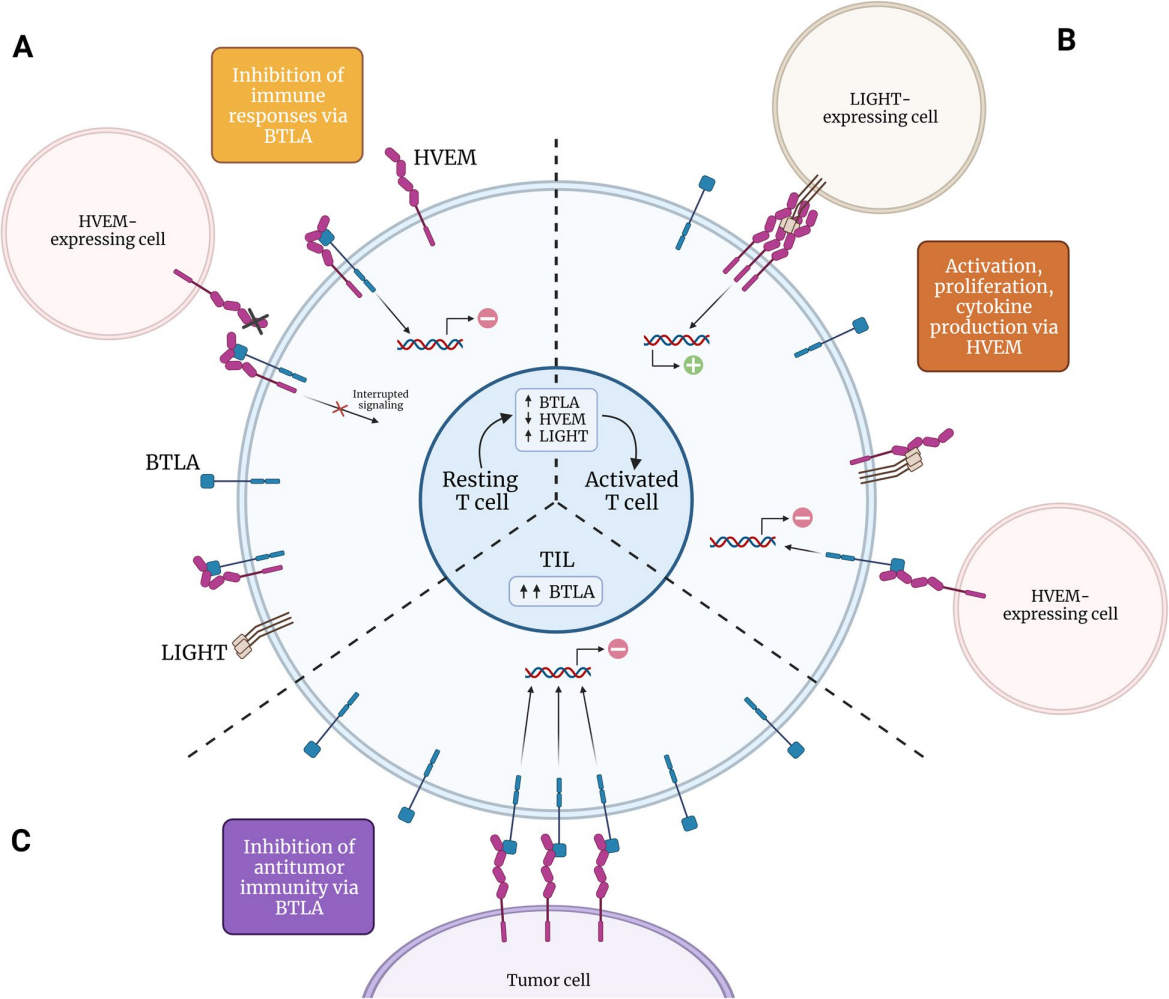
BTLA-HVEM *cis* and *trans* signaling in different settings. **A** In resting T cells, BTLA inhibitory signaling plays a predominant role in *cis* interactions, impeding HVEM-mediated activation. **B** Upon activation, BTLA-HVEM *cis* complex is disrupted thus allowing *trans* interactions between BTLA and HVEM-expressing cells. **C** In the context of cancer, enhanced BTLA expression on tumor-infiltrating lymphocytes increase *trans* interaction and leads to the inhibition of T cell-mediated antitumor responses

In this context, ICB is considered a major breakthrough in the fight against cancer due to its capacity to revert immune exhaustion, and reinvigorate antitumor responses. Noteworthy, ICB is considered a major breakthrough in the fight against cancer. Noteworthy, ICB-based therapies with mAb targeting CTLA-4 or PD-1/PD-L1 promote long-lasting or even curative responses in approximately 20–30% of patients [64]. Yet, a significant percentage of patients do not respond to these immunotherapies, bringing to light the urge to investigate novel potential targets among the broad spectrum of inhibitory receptors that regulate the immune response. In the context of cancer, the immunoregulatory function of HVEM and its ligands, especially BTLA due to its inhibitory nature, is altered, hence contributing to the dysregulation of antitumor immunity (Fig. 3).

Dysregulation of the BTLA/HVEM axis has been described in a plethora of solid tumors and hematological malignancies. In hepatocellular carcinoma, increased percentage of tumor-infiltrating PD-1+ BTLA+ CD4+ T lymphocytes was observed when compared to those from tumor-free regions from the same patient, pointing out a role of the tumor microenvironment (TME) in this dysregulation [17]. Similarly, elevated surface levels of BTLA on infiltrating CD4 + and CD8+ T lymphocytes in bladder cancer were correlated with poor outcomes [65]. Likewise, patients with non-small cell lung cancer (NSCLC) negative for BTLA and PD-L1 expression exhibited a better prognosis [66]. Moreover, HVEM was found to be overexpressed in 18.6% of NSCLC patients, mainly those with lymph node metastasis and advanced stage [67]. These results are consistent with those obtained in a study with 136 patients with gastric cancer, where high BTLA and HVEM levels were associated with lymph node metastases and diminished overall survival [68]. *BTLA* gene expression in whole blood was augmented in patients with colorectal cancer compared to healthy individuals, which in turn was correlated with the expression of other checkpoints such as LAG-3 or PD-1, as well as lower overall survival [69]. On the other hand, in silico analysis of HVEM levels in endometrial cancer unveiled that low mRNA expression was associated with poor prognosis. Besides, in vitro* TNFRSF14* KO showed that this association with decreased survival could be related, at least in part, to enhanced migration and promotion of epithelial-mesenchymal transition (EMT) [70]. These results contrasted with those obtained for melanoma. Using immunohistochemistry and flow cytometry, Malissen et al*.* demonstrated that high HVEM levels were associated with poor prognosis in patients with metastatic melanoma, as well as in other tumors, such as breast and gastric cancer [68, 71–74]. These results were validated by bioinformatic analysis of data from The Cancer Genome Atlas (TCGA) [75]. Multispectral immunofluorescence assays revealed that BTLA+ CD8+ T cells were physically related to HVEM+ tumor cells, thereby suggesting that HVEM molecules on melanoma cells inhibit infiltrating T cells via BTLA [75, 76]. This fully agrees with the results obtained in another independent study in melanoma patients, where it was observed that BTLA expression was reduced during differentiation of CD8+ T lymphocytes to effector cells, whereas in vivo expression was maintained in those lymphocytes specific for melanoma tumor antigens hindering their antitumor activity [77].

Despite BTLA-mediated inhibition of immune responses, this receptor can, promote positive signals through its Grb2 domain. Two studies on adoptive T-cell therapy in murine melanoma models described that BTLA- CD8+ T lymphocytes were not able to control tumor development in vivo, while their BTLA+ counterpart exhibited antitumor capacity, as well as greater survival and resistance to apoptosis [78, 79]. HVEM binding to BTLA induced inhibition of CD8+ T lymphocyte proliferation as well as cytokine production, although AKT signaling was also triggered protecting T lymphocytes from apoptosis [79].

### BTLA/HVEM axis in hematological cancers

Various studies have highlighted the importance of the BTLA/HVEM axis in tumor development, immunosuppression and prognosis. High HVEM and low BTLA mRNA levels in tumor cells from patients with follicular lymphoma (FL) and diffuse large B-cell lymphoma (DLBCL) were associated with shorter time-to-treatment, overall survival, and increased risk of transformation from FL to DLBCL [80, 81]. Contrarily, other works support a tumor suppressor role for HVEM in these hematological tumors. Loss-of-function mutations are often detected in *TNFRSF14* gene that encodes this receptor, which are among the most frequently mutated genes in FL and DLBCL (up to 50% of cases), and associated with poor prognosis [80, 82–85]. Michael Boice et al*.* sustain that HVEM inactivation has a direct pro-tumoral role in lymphoma cells, and also influences the TME. This hypothesis might have a simple explanation based on BTLA signaling. First, BTLA ITIM domains not only interact with SHP, but also with CD79, a typical BCR signaling mediator [86]. As a consequence, *trans* HVEM-BTLA interaction among adjacent tumor cells could lead to BTLA-mediated transduction of negative signals to partially hinder tumor development, leading to a kind of contact inhibition and preventing tumor proliferation. Thus, disruption of the interaction between HVEM and BTLA through inactivating mutations or downregulation of these immune checkpoints provides a mechanism for stimulating BCR-related mitogenic signals in B-cell lymphoma cells [82]. This hypothesis about the tumor suppressive role of HVEM in lymphomas is supported by subsequent studies*.* Follicular helper T lymphocytes by decreasing BTLA surface expression, promoted the upregulation of the anti-apoptotic protein BCL-2 (via CD40L) in B germinal centers, favoring tumor growth [87, 88]. Therefore, according to these data, further studies are required to clarify more precisely the role played by the BTLA/HVEM axis in FL and DLBCL, either as a tumor suppressor or progression promoter by NF-κB pathway activation.

Analysis of BTLA expression in 253 samples from patients with non-Hodgkin's lymphomas from germinal centers, including DLBCL, mantle cell lymphoma (MLC), marginal zone lymphoma, Burkitt's lymphoma and chronic lymphocytic leukemia (CLL) revealed that the latter displays the highest expression levels, suggesting that BTLA and HVEM expression on tumor cells from germinal centers could serve as a marker to distinguish between different types of non-Hodgkin's lymphomas [88]. CLL not only exhibits exacerbated BTLA levels, HVEM expression is also deeply dysregulated in this tumor. Moreover, higher *TNFRSF14* levels in leukemic cells were correlated with shorter overall survival [89]. In line with this, BTLA expression is significantly augmented in CD4+ and CD8+ T lymphocytes, as well as in NK cells. Importantly, increased levels of this inhibitory immune checkpoint on CD4+ T lymphocytes (but not CD8+) and NK cells correlated with shorter time to treatment in patients with CLL, thus suggesting an important role of BTLA dampening antitumor responses [89, 90]. In agreement, Alan G. Ramsay et al*.*, analyzed the expression of BTLA in peripheral blood T lymphocytes from patients with CLL and found significantly higher BTLA levels compared to healthy donors [91].

### Soluble form of BTLA in cancer

It is worth mentioning the existence of a soluble form of BTLA (sBTLA), as a consequence of alternative splicing [92]. Even though its role remains as of yet to be clarified, several studies have postulated this molecule as a prognostic factor or a predictor of treatment response. In solid tumors, elevated levels of sBTLA in sera have been associated with decreased survival and aggressive disease in prostate and ovarian cancer, pancreatic adenocarcinoma, clear cell renal cancer, and hepatocellular carcinoma, among others [71, 93–96]. Circulating sBTLA levels have also been detected in patients with CLL [89, 97]. Indeed, increased sBTLA levels were associated with aggressive behavior and decreased time to treatment in these patients [89]. Noteworthy, further analyses of plasma sBTLA levels in patients with various solid tumors before undergoing ICB therapy brought to light that this molecule, beyond being an independent prognostic factor for survival or disease severity, may also serve as a valuable predictive tool for response to ICB-based therapy [98].

### BTLA blockade as a target for cancer immunotherapy

Blockade of the inhibitory signaling pathway mediated by the BTLA/HVEM axis using antagonistic mAbs has been proposed as a potential therapeutic approach in multiple cancers. As an example, BTLA is overexpressed in CD8+ T lymphocytes specific for the tumor antigen NY-ESO-1 in melanoma. In fact, BTLA signaling disruption in vitro increased the proliferation and cytokine production in tumor-specific CD8+ T lymphocytes. Further, this approach showed a cooperative effect promoting T cell activity by blocking simultaneously other immune checkpoints, including TIM-3 and PD-1 [99]. Thus, combined BTLA and PD-1 blockade led to increased T lymphocyte activation, cytotoxicity, and cytokine production in response to allogeneic dendritic cells in vitro, unveiling the potential benefit of this combination [100]. Similar synergistic effects were achieved by combination with chemotherapy in ovarian carcinoma, where BTLA expression was related to poor outcome. BTLA blockade together with the chemotherapeutic agent paclitaxel significantly reduced tumor size and improved survival compared to both monotherapies in murine models of ovarian carcinoma. Furthermore, increased percentage of activated CD8+ and CD4+ T lymphocytes, higher levels of proinflammatory cytokines such as IL-12 or IFN-γ, as well as a reduction of various immunosuppressive cytokines (i.e. IL-10, IL-6 and TGF-β) were also observed by BTLA disruption [101].

Despite several studies strongly support the inhibitory and prognostic value of BTLA and HVEM in hematological cancers, there is scarce information to date about the antitumor effects associated to disruption of BTLA/HVEM axis. In-depth functional characterization is fundamental for safe implementation of BTLA blockade strategies into future clinical trials. As previously mentioned, BTLA expression is upregulated on the surface of CD4+ and CD8+ T lymphocytes as well as NK cells from patients with CLL, and negatively impacted patient´s outcome. Treatment with an anti-BTLA agonistic antibody decreased IL-2 and IFN-γ production by cytotoxic lymphocytes from CLL patients, whereas BTLA blockade restored cytokine production. Moreover, anti-BTLA blocking mAb promoted NK and T cell-mediated cytotoxicity, antibody-dependent cytotoxicity (ADCC) and was able to effectively eliminate leukemic cells ex vivo [89, 90].

Since reduced expression or deleterious mutations of HVEM was found to promote lymphomagenesis and tumor development in patients with FL and DLBCL, this molecule has been proposed as a possible therapeutic target [82]. In this context, a soluble form of HVEM (sHVEM) might be effective to restore tumor suppression upon binding to BTLA on cancer cells. In agreement, CD19-targeting and sHVEM-producing CAR-T cells were more effective than those exclusively based on CD19-CAR-T in xenograft murine lymphoma models, since sHVEM released locally within the tumor avoided undesired systemic immunosuppressive effects [82].

**Fig. 4:**
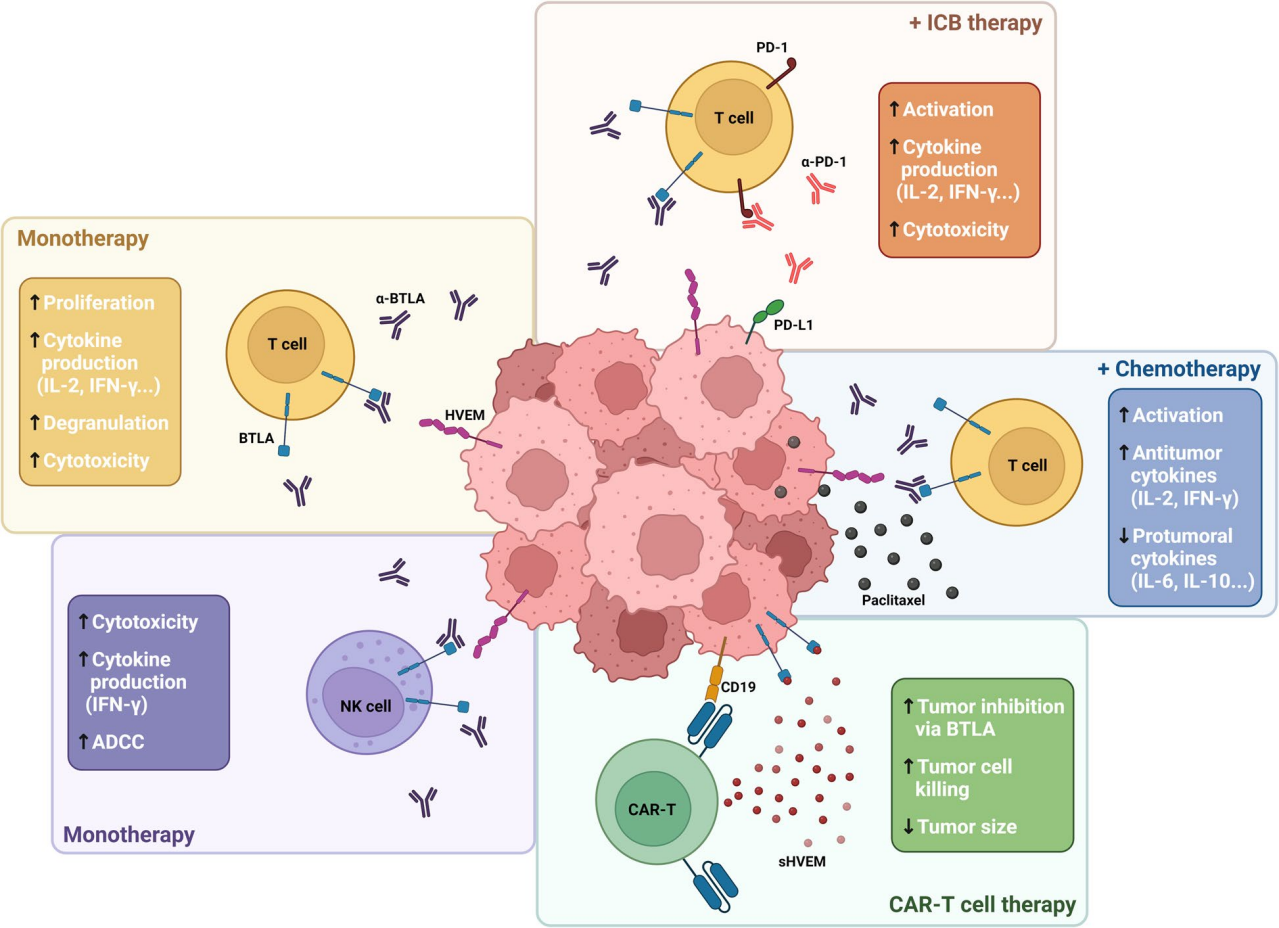
BTLA/HVEM axis as a target for cancer immunotherapy. Multiple approaches are being developed at pre-clinical and clinical levels, including monoclonal antibodies (in monotherapy or combination with anti-PD-1 or chemotherapeutic agents) and sHVEM-producing CAR-T cells

Allogeneic hematopoietic stem cell transplantation (allo-SCT) is a common treatment (sometimes the only curative option) in multiple hematological cancers. This approach enhances antitumor responses mediated by alloreactive T lymphocytes directed against minor histocompatibility antigens (mHAgs) [102, 103]. Increased BTLA expression was observed in mHAg-specific CD8+ T cells compared with memory effector CD8+ T cells in patients with acute myeloid leukemia and multiple myeloma, as well as constitutive expression of HVEM in mHAgs tumor cells. Ex vivo BTLA blockade increased proliferation, cytokine production and degranulation of mHAg-specific CD8+ T lymphocytes. On this basis, targeting BTLA inhibitory signaling poses as a promising strategy to enhance T cell reactivity during allo-SCT [18]. Therapeutic alternatives targeting BTLA/HVEM axis currently explored in cancer are summarized in Fig. 4.

## Clinical development of BTLA-targeted therapeutics

In 2019, it was approved the world´s first-in-class anti-BTLA blocking mAb tifcemalimab (icatolimab, TAB004/JS004, Junshi Biosciences), hindering BTLA/HVEM interaction. Tifcemalimab/icatolimab is an IgG_4_ mAb, currently under clinical development in several solid tumors and hematological malignances (summarized in Table 1). However, little data is available up to date regarding the clinical efficacy of this immunotherapeutic agent. In a phase Ia dose-escalation study (NCT04137900) with 25 patients with advanced solid tumors (median of 4 prior lines of therapy), including 15 patients who progressed upon anti-PD-1/PD-L1 therapy, tifcemalimab/icatolimab demonstrated a good safety profile and preliminary clinical efficacy (one partial response and six stable disease among 19 evaluable patients with a median follow-up of 32 weeks). Interestingly, joint expression of CD8 and HVEM has been proposed as a potential biomarker for positive response to BTLA blockade; however, there is scarce data available and extensive validation studies are required [104]. Forty-three patients with extensive-stage NSCLC refractory to prior therapies (including 14 patients who underwent anti-PD-1/PD-L1-based treatment) were enrolled in a phase I/II study (NCT05000684) exploring dual BTLA and PD-1 blockade. Despite preliminary data suggesting that tifcemalimab in combination with PD-1/PD-L1 axis blockade is well tolerated with 70.0% of the responses ongoing (median follow-up, 12.1 weeks), the median duration of response was not reached at the cut-off date [105].

**Table 1:** Current clinical trials targeting the BTLA/HVEM axis

Clinical trial	Disease	Therapy	Phase	Status
NCT04773951	Melanoma, renal carcinoma and urothelial carcinoma	Monotherapy and tifcemalimab/icatolimab + toripalimab (anti-PD-1)	Phase I	Recruiting
NCT05000684	Advanced lung cancer	Monotherapy and tifcemalimab/icatolimab + toripalimab (anti-PD-1)	Phase I	Recruiting
NCT05427396	Liver cancer, esophageal squamous cell carcinoma, gastric adenocarcinoma, cervical cancer, colorectal cancer	Tifcemalimab/icatolimab + toripalimab (anti-PD-1)	Phase I	Recruiting
NCT04929080	Head and neck cancer and nasopharyngeal carcinoma	Monotherapy and tifcemalimab/icatolimab + JS001 (anti-PD-1)	Phase I/II	Recruiting
NCT04477772	Recurrent/Refractory malignant lymphoma	Monotherapy and tifcemalimab/icatolimab + JS001 (anti-PD-1)	Phase I	Recruiting
NCT04137900	Advanced unresectable solid tumor and metastatic solid tumor	Tifcemalimab/icatolimab + toripalimab (anti-PD-1)	Phase I	Recruiting
NCT05664971	Advanced lung cancer	Tifcemalimab/icatolimab + JS001 (anti-PD-1) alone or in combination with chemotherapy	Phase I/II	Recruiting
NCT05891080	Stage III resectable or potentially resectable non-small cell lung cancer	Tifcemalimab/icatolimab + toripalimab (anti-PD-1) + chemotherapy + surgery	Phase II	Not recruiting yet
NCT05789069	Advanced renal cell carcinoma, melanoma, non-small cell lung cancer, gastric cancer, and colorectal cancer	HFB200603 (anti-BTLA) alone or in combination with tislelizumab (anti-PD-1)	Phase 1a/1b	Recruiting

Regarding hematological malignancies, a phase I study (NCT04477772) using tifcemalimab/icatolimab as monotherapy or in combination with the anti-PD-1 blocking mAb toripalimab is currently ongoing, with quite promising preliminary results. A total of 48 patients has been enrolled, including 20 non-Hodgkin´s and 28 Hodgkin´s lymphomas. All patients were heavily treated, with a median of 4 prior lines, including 32 patients who underwent anti-PD-1/PD-L1 therapy. With a median follow-up of 31.3 weeks, a total of one partial response (follicular lymphoma) and seven stable disease were observed in the tifcemalimab/icatolimab monotherapy cohort (*n*=22, including 11 individuals refractory to prior anti-PD-1/PD-L1 therapy). Among 12 evaluable patients with Hodgkin´s lymphoma, one patient treated with dual BTLA/PD-1 blockade showed complete response, 4 of them partial response and 5 stable disease (including 7 individuals refractory to prior anti-CD30 therapy) [106, 107]. Interestingly, similar to solid tumors, a trend of correlation between high HVEM expression and clinical response was observed. Overall, treatment with tifcemalimab/icatolimab was well-tolerated and demonstrated preliminary clinical efficacy in solid tumors and lymphomas, thus encouraging further investigation of BTLA/HVEM axis as a promising target for immunotherapy.

## Concluding remarks and future directions

ICB-based therapies have become an essential treatment option for a considerable number of tumors, despite the disappointing results obtained in others. On this basis, development of novel strategies for effectively targeting immune checkpoints represents a major milestone in cancer treatment to improve clinical efficacy and patient benefit by expanding the therapeutic spectrum.

As reviewed herein, recent accumulative evidence is shedding light on the key role of BTLA and HVEM in tumor immune escape. In good agreement, dysregulation of BTLA/HVEM axis has been correlated with prognosis in both solid and hematological cancers. Moreover, circulating sBTLA has been revealed as a blood-based predictive biomarker of time to treatment and response to ICB-based therapies in various malignancies. Hence, BTLA/HVEM axis emerges as a novel and promising target for cancer immunotherapy. Accordingly, mounting preclinical data strongly support the function of BTLA dampening T lymphocyte and NK cell-mediated antitumor responses, which could be restored by employing blocking antibodies thereby subsequently triggering increased proliferation, cytokine production and cytotoxicity. As a next step into clinical application, the anti-BTLA blocking mAb tifcemalimab/icatolimab has entered into various ongoing phase I/II clinical trials with very encouraging results so far. Thus, tifcemalimab/icatolimab has demonstrated preliminary efficacy and safety profile in both solid and hematological malignancies. It is worth mentioning that, along with preclinical evidence, combination of anti-BTLA and current anti-PD-1/PD-L1 therapies has demonstrated promising results, even in those patients refractory to previous ICB monotherapy.

Besides cancer immunotherapy, it is worth mentioning that BTLA/HVEM axis is also likely to emerge as a critical player in other diseases, including sepsis [92, 108–110], neuroinflammation [111], infectious [112–114] and autoimmune diseases [115–119]. Three clinical trials using agonistic anti-BTLA antibodies (LY3361237, Stanford University and Eli Lilly and Company and ANB032 from AnaptysBio) are currently undergoing in patients with systemic lupus erythematosus (NCT05123586), Sjogren's syndrome (NCT05781451), and severe atopic dermatitis (NCT05935085). Moreover, agonistic anti-BTLA antibodies have been proposed as a potential therapy to ameliorate GvHD resulting from allogeneic bone marrow transplantation or to prolong allograft survival in models of renal or cardiac transplantation [120–125]. Nevertheless, studies targeting BTLA and HVEM are currently under preclinical development. In the framework of infectious diseases, preclinical data indicate that ICB could pose a highly relevant option in tuberculosis, malaria and HIV or hepatisis B infection [126, 127]. Nonetheless, despite several ongoing clinical trials using ICB-based therapies, as of yet, none of them targets BTLA/HVEM axis [126, 128].

Nevertheless, it should be noted the complexity of HVEM signaling, which is part of an intricate network constituted by *cis* and *trans* interactions with distinct binding partners. As a consequence, it appears reasonably essential to deepen understanding of the functional interactome landscape of BTLA/HVEM axis in different cancer settings in order to establish individual therapeutic approaches for adequate patient stratification and safe clinical testing and to ultimately achieve the best outcomes for cancer patients.

In conclusion, compelling evidences indicate that BTLA/HVEM axis holds immense potential for future development of cancer immunotherapy strategies. As ongoing research are shedding new light on the complexity and intricate signaling interactions of BTLA/HVEM axis, therefore it poses as major challenges the identification of reliable predictive biomarkers to guide/monitor treatment response, and to delve into the optimization of combination therapies (mainly anti-PD-1/PD-L1-based therapies). Beyond the oncology field, the implication of BTLA/HVEM axis in transplantation, infections and autoimmune diseases offers promising avenues for investigation and, more importantly, opens up wide and great perspectives for future clinical application.

## Data Availability

Not applicable.
